# Evaluation of Hospital Admission Status for Emergency Department Patients Seen for Chronic Obstructive Pulmonary Disease Exacerbation: A Retrospective Observational Study

**DOI:** 10.31486/toj.19.0121

**Published:** 2021

**Authors:** Hayden L. Smith, Corey S. Ellis

**Affiliations:** ^1^ Department of Medical Education Services, UnityPoint Health–Des Moines, Des Moines, IA; ^2^ Department of Internal Medicine, Carver College of Medicine, University of Iowa, Iowa City, IA

**Keywords:** *Anxiety*, *depression*, *emergency service–hospital*, *patient admission*, *pulmonary disease–chronic obstructive*

## Abstract

**Background:** Chronic obstructive pulmonary disease (COPD) is a common and preventable condition. The disease accounts for a large economic burden in the US health care system. Better control and prevention of COPD exacerbations can help prevent presentations to already-crowded emergency departments (EDs) and hospitals. The objective of our study was to identify variables associated with hospital admission status in ED patients presenting with COPD exacerbation.

**Methods:** We conducted a retrospective observational study of patients seen at 1 of 3 US EDs from 2012 to 2014 with a primary diagnosis related to COPD exacerbation. Hospital admission status was modeled using patient characteristic data via adaptive least absolute shrinkage and selection operator logistic regression. Study results are presented as adjusted odds ratios with 95% CIs. Planned post hoc model dependency and external data sensitivity analyses were conducted.

**Results:** The study sample included 1,165 unique patients with COPD with an ED encounter related to exacerbation at 1 of the 3 reviewed hospitals. Approximately half of these patients had a hospital admission. Variables inversely associated with an admission included oxygen saturation and number of prior ED encounters for COPD exacerbation. Variables positively associated with admission were initial ED heart rate, patient age, and documented comorbidities of anxiety and/or depression. These mental health comorbidities had the strongest association with admission status.

**Conclusion:** Understanding the characteristics of admitted patients may help direct resources and outpatient services to prevent encounters. Of note, the study revealed mental health variables as being strongly associated with admission status.

## INTRODUCTION

Chronic obstructive pulmonary disease (COPD) is a common and preventable condition. In 2010, the estimated number of COPD cases was 384 million, with a global prevalence of 11.7%.^1^ COPD is a leading cause of morbidity and is the fourth leading cause of death in the world.^2,3^ Driving factors related to the burden of COPD are increasing rates of smoking in developing countries and aging populations in developed countries.^4^

COPD is associated with a large economic burden in the US health care system. According to Guarascio et al, in 2010 the estimated direct cost of COPD in the US was $30 billion, with indirect costs of $20 billion.^5^ Singh and Yu reported that the average emergency department (ED) charge was $2,812, and the hospitalization charge was $29,043 for COPD-related illness in 2012.^6^ US data from 2015 to 2016 showed a marked increase in the percentage of adults with a COPD diagnosis receiving ED services.^7^ The cost burden has also been reported in other areas of the world. In 2019, Kirsch et al reported that hospital admissions for COPD accounted for more than 50% of total direct health care costs in a large German sample.^8^

The identification of risk factors associated with exacerbations has been an area of interest for researchers. Better control and prevention of exacerbations can help prevent presentations to already crowded EDs and hospitals as well as address the significant financial weight associated with these visits. While most COPD exacerbations are managed outside of the hospital, the percentage of COPD exacerbations that require hospitalization present an opportunity for health care improvement. Studies have identified risk factors for COPD exacerbations such as decreased forced expiratory volume, anxiety, depression, duration of disease, sex, age, socioeconomic level, and previous admission for exacerbation.^9-14^ Patient age and number of prior exacerbations have been commonly reported predictors of a future exacerbation. Biomarkers, specifically serum/sputum eosinophil levels and C-reactive protein, have also been used to predict exacerbations.^15,16^

Compared to risk factors for COPD acute exacerbation, markedly less research has been done on factors related to hospitalization for COPD exacerbation. Parshall and Doherty (2006) found that a greater heart rate in the ED was predictive of a hospital admission.^17^ Additionally, risk factors such as time of presentation after onset of symptoms, prior admissions for COPD exacerbation, level of dyspnea, and presence of comorbid conditions have been reported as predictive indicators of hospitalization for COPD exacerbations.^18-21^ The aim of our study was to expand on existing research in identifying targetable variables associated with admission status in a sample of US patients with COPD.

## METHODS

We conducted a retrospective observational study of data from 3 community teaching hospital EDs located in the same US Midwestern city. All facilities were within the same health care system and used the same electronic health record (EHR). The study received approval from the institutional review board of record for all 3 study hospitals, and the study was granted a waiver of consent.

Eligible patients had an ED encounter between March 2012 and September 2014, with a primary diagnosis related to COPD exacerbation (*International Classification of Diseases-9-Clinical Modification*: 491.2, 491.21, 491.22, 491.8, 491.9, 492.8, 494, 494.1, 496).

All study data were electronically pulled from the EHR. Candidate categorical variables considered for model inclusion were sex (male/female), race (white, yes/no), weekday (yes/no), open clinic or urgent care clinic in health system at time of ED arrival (yes/no), documented primary care provider (PCP, yes/no), secondary diagnosis of depression (yes/no), and secondary diagnosis of anxiety (yes/no). Candidate quantitative variables were age (years), number of prior ED encounters for COPD exacerbation in the health system during the study period, and initial ED vital data (ie, blood oxygen saturation percentage, respiratory rate breaths per minute, and heart rate bpm).

### Data Analysis

After a preliminary examination of study data, the low rate of missing data was considered missing completely at random, and complete case data were used in analyses. Variable selection was conducted via adaptive least absolute shrinkage and selection operator (adaLASSO) multiple logistic regression to address the bias-variance tradeoff in model development. Estimates for selected variables were calculated using a postselective inference correction and represented an SD change in the covariate. Categorical variables had their odds ratios calculated on their original binary scale using estimates from a multiple logistic regression. Estimates are presented as adjusted odds ratios (aOR) with 95% CIs.

A sensitivity analysis for model dependency regarding variable selection was conducted by additionally fitting a planned post hoc nonparametric model. The generated ranking list of variable importance from that model was contrasted with variables selected from the adaLASSO model. External sensitivity analyses were also conducted to examine estimate robustness to omitted variable bias. In particular, E-value estimates were calculated based on confounder(s) equi-associated with the independent and dependent variable large enough to nullify the lower CI for the estimates. Statistical and sensitivity analyses were conducted using R 3.4.4 (R Foundation for Statistical Computing) and SAS statistical software, version 9.4M3 (SAS Institute, Inc). A supplemental analysis document is available that contains details on the examination of data missingness, model selection, and sensitivity analyses. To obtain a copy of the document, email your request to ocjournal@ochsner.org.

## RESULTS

We identified 1,317 unique patients with at least one eligible ED encounter for COPD exacerbation at the 3 reviewed hospitals during the study period. Of these patients, 1,165 (88.5%) had complete data for all variables and were used as the complete case dataset. Patient characteristics for this study sample are presented in Table 1. Descriptive statistics revealed an average patient age of 67 years. Patients were 40.9% male and 94.0% white, and 54.5% were admitted to the hospital. Average ED vitals for patients were heart rate of 94 bpm, respiratory rate of 22 breaths per minute, and 93% blood oxygen saturation. Most patients (90.8%) had a documented PCP, 73.6% had no prior ED encounter for COPD exacerbation during the study period at one of the study hospitals, 73.8% were seen on a weekday, and 67.3% were seen at a time when clinics or urgent care clinics within the health system were open. Regarding depression and anxiety, 12.8% of patients had a secondary diagnosis of depression, 12.8% had a secondary diagnosis of anxiety, and 4.0% had both diagnoses. The mean length of stay for the 635 admitted patients was 4 days (SD=5, median 3, interquartile range 3-5).

**Table 1.** Characteristics of Emergency Department (ED) Encounters for Chronic Obstructive Pulmonary Disease Exacerbation, n=1,165

Variable	Value
Hospital admission	635 (54.5)
Age, years, mean ± SD	67 ±13
Male	476 (40.9)
White race	1,095 (94.0)
Heart rate, bpm, mean ± SD^a^	94 ± 19
Respiratory rate, breaths per min, mean ± SD^a^	22 ± 5
Blood oxygen saturation, %, mean ± SD^a^	93 ± 5
Documented primary care provider	1,058 (90.8)
Prior ED encounters for exacerbation^b^	
0	857 (73.6)
1	191 (16.4)
2	57 (4.9)
≥3	60 (5.1)
Mean ± SD	0.5 ± 1.4
Weekday arrival	860 (73.8)
Clinic or urgent care clinic open	784 (67.3)
Secondary diagnosis of depression	149 (12.8)
Secondary diagnosis of anxiety	149 (12.8)

^a^Initial ED values.

^b^Prior ED encounter within study period at any of the 3 study hospitals.

Note: Data are presented as n (%) unless otherwise indicated.

Descriptive information for patients stratified by admission status is presented in Table 2. Of note, admitted patients tended to be older, had higher heart and respiratory rates, and had lower blood oxygen saturation. A higher percentage of patients with a documented PCP were admitted, while a lower percentage of patients with prior COPD encounter(s) were admitted. Patients with a documented secondary diagnosis of depression or anxiety had higher prevalence of admission.

**Table 2.** Characteristics of Emergency Department (ED) Encounters for Chronic Obstructive Pulmonary Disease Exacerbation Stratified by Admission Status, n=1,165

Variable	Admitted to Hospital n=635 (54.5)	Not Admitted to Hospital n=530 (45.5)
Age, years, mean ± SD	69 ± 12	65 ± 13
Male	245 (38.6)	231 (43.6)
White race	610 (96.1)	485 (91.5)
Heart rate, bpm, mean ± SD^a^	97 ± 20	91 ± 17
Respiratory rate, breaths per min, mean ± SD^a^	23 ± 6	22 ± 5
Blood oxygen saturation, %, mean ± SD^a^	92 ± 6	94 ± 4
Documented primary care provider	604 (95.1)	454 (85.7)
Prior ED encounters for exacerbation^b^		
0	489 (77.0)	368 (69.4)
1	3 (0.5)	2 (0.4)
2	22 (3.5)	35 (6.6)
≥3	121 (19.1)	125 (23.6)
Mean ± SD	0.5 ± 1.5	0.6 ± 1.3
Weekday arrival	489 (77.0)	371 (70.0)
Clinic or urgent care clinic open	444 (69.9)	340 (64.2)
Secondary diagnosis of depression	131 (20.6)	18 (3.4)
Secondary diagnosis of anxiety	121 (19.1)	28 (5.3)

^a^Initial ED values.

^b^Prior ED encounter within study period at any of the 3 study hospitals.

Note: Data are presented as n (%) unless otherwise indicated.

Determined variables associated with a hospital admission selected from the model building process included heart rate, blood oxygen saturation, secondary diagnosis of anxiety, secondary diagnosis of depression, age, and number of prior COPD ED encounters. These variables explained 74.75% of admission status (Figure). Estimates revealed patients with a secondary diagnosis of depression or anxiety had a respective 6.1 (95% CI, 3.7-10.1) and 3.5 (95% CI, 2.2-5.7) times greater adjusted odds of hospital admission. An SD increase in initial ED heart rate was associated with a 1.4 (95% CI, 1.1-1.6) times greater adjusted odds of admission while an SD decrease in blood oxygen saturation was associated with a 1.3 (95% CI, 1.4-1.8) times greater adjusted odds of admission. Patient likelihood of being admitted increased with age, with an SD increase associated with an aOR of 1.4 (95% CI, 1.3-1.6). Also of note, adjusted odds of admission decreased with the number of prior ED encounters for COPD exacerbation. An SD decrease in the number of past encounters was associated with a 1.25 (95% CI, 1.04-1.36) times higher aOR of admission.

**Figure.**
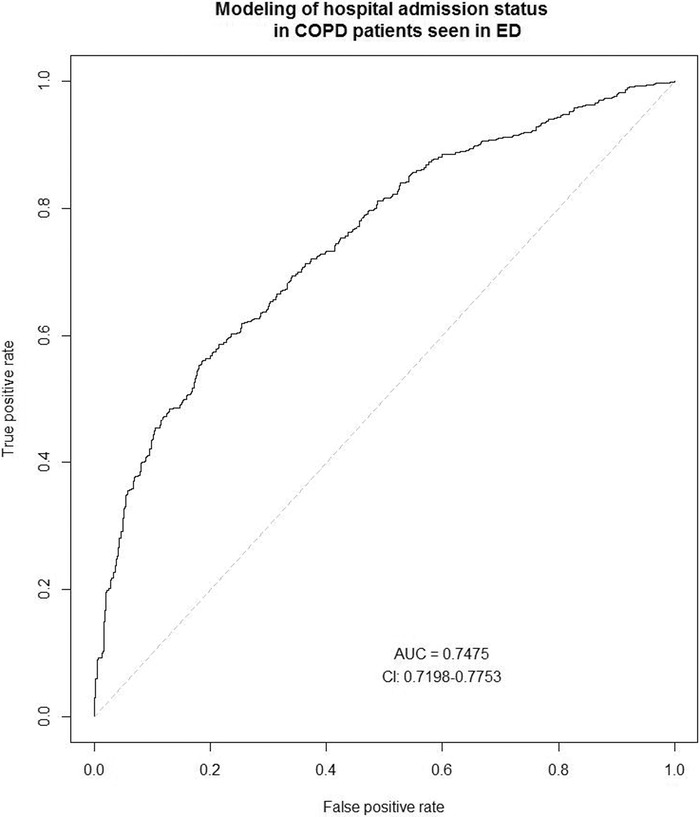
**Receiver operator characteristic curve for the set of nonzero coefficients selected via adaptive least absolute shrinkage and selection operator (adaLASSO) model for variables associated with hospital admission status in patients seen in the emergency department (ED) for chronic obstructive pulmonary disease (COPD) exacerbation (n=1,165). Selected variables included heart rate, blood oxygen saturation, depression, anxiety, age, and prior ED encounter for COPD exacerbation during study period.**

Sensitivity analysis for variable selection because of model dependency revealed no substantive differences in variable selection when a nonparametric model was used. In particular, the generated variable list from the nonparametric model ranked the same variables low that were dropped out of the adaLASSO model. Generated E-values for estimates of admission status were also calculated and are presented as a figure in the supplemental analysis document (available by request by emailing ocjournal@ochsner.org). Of note, an unknown confounder(s) equi-associated with the independent and dependent variables with a relative risk of 2.08, 1.43, 6.86, 3.82, 2.45, or 1.24 for blood oxygen saturation, heart rate, depression, anxiety, age, and number of prior ED encounters, respectively, would be required to nullify their association with admission status.

## DISCUSSION

We reviewed more than 1,000 ED encounters for COPD exacerbation from 3 hospitals. Results revealed that initial ED vital data and diagnoses of mental health comorbidities were associated with hospital admission. In addition, patient age and number of prior ED encounters were also noted to be associated with admission.

The association between age and hospitalization is corroborated by other study findings.^20,22,23^ The association between patient heart rate and hospitalization is similar to results from a study examining heart rate as a predictive variable of ED disposition in patients with COPD.^17^ In that study, an initial heart rate ≥106 bpm was associated with hospitalization, with a reported accuracy of 0.67, sensitivity of 0.61, and specificity of 0.68.^17^ The association between a lower initial blood oxygen saturation and increased relative odds of hospital admission has also been documented in other studies.^16,20,24^ Notably, other studies showed an association between respiratory rate and hospital admission that we did not discern in our study when adjusting for the other vital data within a regularizing model.^20,21,25^

The number of prior ED visits for COPD exacerbation was negatively associated with hospital admission. In general, we may speculate that a history of prior exacerbation would increase patient risk of having future exacerbations. In our study sample, we observed a lower admission status among patients with multiple prior ED encounters for COPD exacerbation compared to patients with no prior ED encounters for COPD exacerbation. Of note, for our study sample, a prior encounter was defined as occurring within the study period and at one of the study centers, which may represent a limited and selective sampling of patient history.^21,22^ These results on prior hospitalization for COPD exacerbation appear paradoxical or even protective for subsequent hospitalization and differ from the findings in other studies.^19,20,26^ Additional research may need to better define the mechanism or phenomena underlying repeated ED use and admission status.

Prior research has investigated comorbid conditions and the hospital admission status of patients with COPD,^7^ while only a few studies have evaluated the presence of anxiety or depression as factors associated with admission.^10,11^

The prevalence of anxiety has been documented as being higher in patients with COPD than in the general population and is estimated to be between 13% and 36%.^27-29^ Also, the risk of COPD exacerbation is increased in patients with comorbid anxiety,^28^ and the comorbidities of anxiety or depression doubled the rates of exacerbation in a study published in 2017.^29^ As of April 2020, no studies have reported an association between anxiety and an initial COPD exacerbation related to hospitalization. Two studies showed an increased likelihood of COPD-related rehospitalization after an initial COPD hospitalization in patients with anxiety compared to patients without anxiety.^30,31^ Further investigation is warranted to explore the correlation between anxiety and COPD-related hospitalization to examine ways to potentially address and better understand possible macro and physiological relationships.

Our study revealed that patients with comorbid depression had an increased likelihood of being admitted for COPD exacerbation from the ED. In general, the prevalence of depression in patients with COPD is higher than in the general population and estimated to be between 12% and 23%,^28,29^ with one study reporting 41%.^32^ A meta-analysis showed that depression was present in 25% of patients with COPD compared to <12% in controls.^33^ Only a few papers have commented on the association between depression and risk of COPD-related hospitalization. Fan et al followed 610 patients and found that patients with severe depression had an increase in respiratory hospitalization with an odds ratio of 2.26 (95% CI, 1.3-3.9); however, after adjustment for disease severity, this relationship was no longer present.^32^ A study conducted by Xu et al of a cohort of 491 patients with COPD in China showed that patients with a secondary diagnosis of depression had higher incidence of COPD exacerbations and hospitalization.^28^ A study by Dalal et al showed that patients with COPD and comorbid depression were 60% more likely to be hospitalized.^34^ Several explanations have been posited about a possible link between depression and COPD. One theory is that depression-related changes in major immune classes may predispose patients with COPD to viral/bacterial infections that in turn trigger COPD exacerbations and possible hospitalizations.^35-37^ Another theory is that patients with COPD and comorbid depression may have difficulty with self-care, including adherence to COPD medication regimens, which may lead to increased exacerbations and potential hospitalizations.^38,39^

### Limitations

Patient anxiety and depression status were identified based on documented secondary diagnoses. No direct systematic diagnostic screenings were performed during hospital encounters for anxiety and depression. In addition, treatment or treatment adherence for anxiety and depression, if chronic, was unknown given the retrospective study design. Similarly, patient disease stage and treatment/maintenance for COPD was not documented, along with antagonists such as smoking or environmental exposures. Also, not available in the medical records was concise information about response treatments (eg, nebulized corticosteroids). Of note, all study patients were from the same health system and geographic area. Whether ED- or medical center–based idiosyncrasies were present in the health system is unknown, as well as unique regional or individual patient characteristics that could have influenced results. An examination of seasonal triggers would have also been beneficial in this study, although such an analysis would have likely required also addressing their variability across years. Last, the observational study design allowed for a risk of omitted variable bias. Sensitivity analyses were conducted to quantify the magnitude of a lurking variable large enough to negate results.

## CONCLUSION

COPD is a leading contributor to morbidity and economic burden. Hospital admission status may serve as a proxy marker for the appropriateness of an ED encounter. As a result, understanding predictors of COPD-related hospitalizations through the ED may provide information to help determine and direct resources as well as outpatient services. This study contributes to the literature by confirming variables and presenting possible strong associations between mental health and hospital admission status. Understanding these variables may assist in defining factors related to future health care encounters.

## Notes

This article meets the Accreditation Council for Graduate Medical Education and the American Board of Medical Specialties Maintenance of Certification competencies for Patient Care and Medical Knowledge.
